# Measuring Mindreading: A Review of Behavioral Approaches to Testing Cognitive and Affective Mental State Attribution in Neurologically Typical Adults

**DOI:** 10.3389/fpsyg.2017.00047

**Published:** 2017-01-24

**Authors:** Rose Turner, Fatima M. Felisberti

**Affiliations:** Emotion, Cognition and Behavior laboratory (ECOBEL), Department of Psychology, Kingston UniversityLondon, UK

**Keywords:** mindreading, theory of mind, mind perception, social cognition, mentalizing, emotion recognition, review

## Abstract

*Mindreading* refers to the ability to attribute mental states, including thoughts, intentions and emotions, to oneself and others, and is essential for navigating the social world. Empirical mindreading research has predominantly featured children, groups with autism spectrum disorder and clinical samples, and many standard tasks suffer ceiling effects with neurologically typical (NT) adults. We first outline a case for studying mindreading in NT adults and proceed to review tests of emotion perception, cognitive and affective mentalizing, and multidimensional tasks combining these facets. We focus on selected examples of core experimental paradigms including emotion recognition tests, social vignettes, narrative fiction (prose and film) and participative interaction (in real and virtual worlds), highlighting challenges for studies with NT adult cohorts. We conclude that naturalistic, multidimensional approaches may be productively applied alongside traditional tasks to facilitate a more nuanced picture of mindreading in adulthood, and to ensure construct validity whilst remaining sensitive to variation at the upper echelons of the ability.

## Introduction

*Mindreading* describes the ability to attribute mental states to oneself and others, and is essential for predicting behavior (Nichols and Stich, 2003). It comprises cognitive and affective components dissociable at the neural level (Shamay-Tsoory and Aharon-Peretz, 2007; though see Pessoa, 2008), and can be both explicit (deliberate) and implicit (automatic; Heyes and Frith, 2014), expressed via two-systems (Apperly and Butterfill, 2009) and multi-systems cognitive models (Christensen and Michael, 2016). Mindreading is also referred to as *Theory of Mind* (ToM; Wimmer and Perner, 1983) and *mind perception* (Gray et al., 2010). As ToM alludes to an elaborate accumulation of concepts and mind perception minimizes agency, the term mindreading is employed here.

Since Premack and Woodruff (1978) posed the question, “does the chimpanzee have a ToM?”, empirical mindreading research has focused on child development, autism spectrum disorder (ASD) and, more recently, clinical groups, whereas studies featuring neurologically typical (NT) adults are less frequent. There are compelling arguments for investigating adults’ mindreading: mindreading ability changes across the lifespan (Happé et al., 1998; Maylor et al., 2002; Duval et al., 2010) and is positively associated with effective interpersonal relationships (Castano, 2012) and prosocial behavior (Paal and Bereczkei, 2007; Johnson, 2012). Studying adults facilitates the construction of theoretical models, which supports an understanding of mindreading development (Apperly et al., 2009) and identification of diagnostic markers for ASD, clinical and neurodegenerative disorders (e.g., Poletti et al., 2012; Guastella et al., 2013).

Explicit and implicit mindreading abilities appear dissociable in children, ASD and clinical samples (Onishi and Baillargeon, 2005; Senju et al., 2009), but closely related in NT adults (Kanske et al., 2015), hence this paper will focus on explicit measures. Whereas implicit mindreading is measured indirectly (e.g., via eye-gaze), explicit tasks probe deliberate mental state reasoning. A challenge for researchers lies in establishing behavioral tools sensitive to variation at the upper echelons of mindreading, since NT adults tend to perform at ceiling (at or near 100% accuracy) on standard explicit measures.

Mindreading is a multidimensional construct, which has led to inconsistent definitions across the literature (Schaafsma et al., 2015). We aim to cover the empirical ground by addressing: (1) emotion recognition tests [emotion perception reflects a low-level process in affective mindreading (Mitchell and Phillips, 2015)]; (2) cognitive and affective mentalizing tasks measuring attribution of beliefs, intentions, desires, and emotions, respectively; (3) multidimensional measures combining these facets. Rather than present an exhaustive review of the literature, we focus on selected experimental paradigms illustrative of four core approaches: emotion recognition, social vignettes, narrative fiction, and participative interaction, highlighting challenges for use with NT adult samples.

## Emotion Recognition

The ability to recognize emotions precedes affective mindreading (Mitchell and Phillips, 2015). Emotion recognition tests traditionally require participants to identify basic emotions (happiness, sadness, anger, fear, surprise, contempt, and disgust; Ekman and Friesen, 1971, though see Awasthi and Mandal, 2015) presented in photographs or brief video-clips of posed facial expressions. The emotion perception literature has primarily focused on *macroexpressions*: full-face unconcealed expressions lasting more than 0.5 s, however, strong agreement of the basic emotions can result in ceiling effects with NT adults. One approach is to speed up presentation so that stimuli represent *microexpressions* (Ekman and Friesen, 1976), which last up to 0.25 s and are usually fragmentary (appearing on the top or bottom half of the face). Brief presentations can remain on the retina for longer [e.g., Brief Emotion Recognition Test (BART; Ekman and Friesen, 1974)], though this is resolvable by incorporating neutral expressions as forward–backward masks (Matsumoto et al., 2000). Microexpressions are involuntary, tending to signal concealed or altered emotion expressions, so perceiving them likely reflects the advanced capacity to detect deception in real-life interactions (Frank and Svetieva, 2015).

The Reading the Mind in the Eyes Test (*Eyes Test*; Baron-Cohen et al., 2001a), requires participants to attribute the most appropriate mental state term (e.g., “ashamed,” “nervous,” “suspicious,” and “indecisive”) to photographs of the eye-regions of faces. The task probes non-automatic processes (Bull et al., 2008), was designed to detect subtle deficits (Baron-Cohen et al., 1997), and has been applied to a range of domains, including brain studies (Adolphs et al., 2002), dementia (Gregory et al., 2002), and clinical disorders (e.g., Fett et al., 2011). The Eyes Test demonstrates particularly strong predictive power with ASD groups, supporting its validity as a measure of the social cognitive deficits characteristic of ASD: In the original study, performance negatively correlated with Autism Spectrum Quotient scores (Baron-Cohen et al., 2001b), which may be due to the “purity” of the stimuli minimizing the opportunity to depend on alternative (e.g., verbal) cues (cf. Happé, 1995). The Eyes Test is one of few “classic” mindreading tasks sensitive to variation in NT adults, however, it measures emotion recognition rather than mindreading *per se.* This is an important distinction as emotion recognition and other mindreading dimensions can dissociate (Oakley et al., 2016).

Emotion recognition stimuli processed via a single modality [including facial/body images or auditory voice recordings (e.g., Rutherford et al., 2002)] present a specific problem for research with NT adults, and a general issue of ecological validity. Older adults tend to perform poorly compared to young adults on static emotion recognition tests, whilst outperforming them at recognizing continuous emotions in dyadic interactions (Sze et al., 2012). Dynamic stimuli can be used to circumvent problems faced using static images (Biele and Grabowska, 2006; Halberstadt et al., 2011), although both static and dynamic, visual and prosodic affective stimuli lack contextual information (Achim et al., 2013). Therefore, emotion recognition tasks may be most fruitfully applied in conjunction with mental state reasoning measures to facilitate a more comprehensive approach.

## Cognitive and Affective Mentalizing

### Social Vignettes

Cognitive mentalizing entails setting aside one’s own perspective to attribute states to other agents. Both children and adults demonstrate automatic egocentric bias in verbal and visual perspective-taking tasks (e.g., Epley et al., 2004), however, NT adults can partially correct for it (Wang et al., 2014). Belief-attribution, for example, has been shown to be non-automatic in adults (Back and Apperly, 2010). The concept that mindreading ability is indicated by understanding not simply what someone knows, but their *mistaken* beliefs (Dennett, 1978), led to the development of Wimmer and Perner’s (1983) *false-belief task* (FBT), which depicts belief-states through social vignettes. In the traditional object-transfer paradigm, participants must identify a target agent’s mistaken belief about the location of an object, through understanding that the agent lacks knowledge that the object has moved. For example, A wrongly believes that the sweets are in the opaque jar, because they did not witness B move them to the cupboard (first-order); B wrongly believes A will look for the sweets in the jar, unaware that A secretly watched them being moved (second-order).

*False-belief task*s have been applied to child development studies (for a meta-analysis, see Wellman et al., 2001), ASD (Baron-Cohen et al., 1985), psychiatric disorders (Frith and Corcoran, 1996), brain damage (Winner et al., 1998), stroke (Happé et al., 1999), and Alzheimer’s (Le Bouc et al., 2012). As children typically pass first- and second-order FBTs aged 4–5 (Astington and Dack, 2008) and 6–7 (Perner and Wimmer, 1985) respectively, they tend to show ceiling effects with adults. Adaptations for use with NT adults include a version where participants rate the likelihood that protagonist “Sally” will look for an object in various locations (Birch and Bloom, 2007). Participants are privy to the object’s location in one condition, and the task is sensitive to the interference of that knowledge (“reality bias”; Mitchell et al., 1996).

False-belief understanding has become synonymous with mindreading, however, the construct validity of FBTs has been called into question (e.g., Bloom and German, 2000). For example, the *False-Belief Localizer* tool for isolating the neural basis of false-belief representation (Saxe and Kanwisher, 2003; Dodell-Feder et al., 2011), is often referred to as the “ToM Localizer,” yet the neural pattern diverges from meta-analytic accounts of the ToM network (Spunt and Adolphs, 2014). In developmental populations, poor FBT performance may reflect general task demands (Siegal and Beattie, 1991; Sullivan et al., 1994), and some individuals with ASD pass second-order tasks whilst exhibiting real-life social cognitive difficulties (Happé, 1994), suggesting they may recruit compensatory verbal strategies (Happé, 1995) such as knowledge of complement syntax (Lind and Bowler, 2009). Social animation tasks (e.g., Castelli et al., 2000) circumvent this issue, requiring participants to attribute intentions to animated geometric shapes, though they lack the range of epistemological and emotional information present in ecological stimuli.

The computerized *Yoni Test* (Shamay-Tsoory and Aharon-Peretz, 2007) requires integration of visual and verbal cues, and generates both behavioral and neuroimaging data. A series of vignettes feature a central character, “Yoni,” depicted by a simple cartoon “smiley,” and four images of a single category (e.g., faces, animals, and transport) alongside sentences containing blanks. Participants indicate by mouse-clicking the appropriate image, what Yoni is close to, thinks about, loves, does not love, or identifies with (first-order), and whose misfortune Yoni gloats over, whose success Yoni envies, and items Yoni thinks about, has or loves, that another character thinks about, has or loves (second-order). The task entails interpretation of proximity, eye-gaze and facial expressions, and measures response time and accuracy across cognitive, affective and physical (control) trials.

In the original study, success was higher on affective compared to cognitive trials, a finding replicated by Kalbe et al. (2010), who suggested that additional facial expression cues in the affective condition facilitated decision-making (the scoring system does not separate out the emotion recognition dimension). Nonetheless, second-order differences between controls and patients with ventromedial frontal lobe damage were observed only in the affective condition, indicating that cognitive and affective neural systems are partially dissociable (Shamay-Tsoory and Aharon-Peretz, 2007). The Yoni Test has shown sensitivity to variation in NT adults where FBTs have proven insufficient (e.g., Kidd and Castano, 2013), however, the simplistic stimuli may enable participants to form basic object-agent associations rather than engage in mindreading (also a criticism of FBTs; Perner and Ruffman, 2005). The *Why/How Task* (Spunt and Adolphs, 2014) – an alternative approach to linking neuroscientific and behavioral data – prevents the formation of basic associations by asking participants *how* (physical) and *why* (mindreading) questions about human behaviors depicted through photographs. Designed for *f*MRI studies, the Why/How Task also generates reliable behavioral (accuracy and response time) data. Whilst simple social images do not reflect the complexity of real-world mindreading stimuli, they present opportunities to examine the brain basis for behavioral differences between participants.

### Narrative Fiction (Prose)

Naturalistic narrative stimuli allow mindreading targets to be contextually embedded (e.g., Frith and Corcoran, 1996; Saxe and Wexler, 2005), which may inhibit non-mindreading strategies (Happé, 1995). Happé’s (1994) *Strange Stories Task* assessed comprehension of short, naturalistic narratives including joke, lie, appearance/reality, and contrary emotions. The range of narratives proved more sensitive to subtle between-group differences than FBTs, paving the way for more complex narrative-based approaches.

Participants in the Short Story Task (*SST*; Dodell-Feder et al., 2013), read a fictional story about two characters whose romantic relationship breaks down (Hemingway, 2003). It contains first- and second-order mental states and requires synthesis of contextual, verbal and physical information. Semi-structured questions probe explicit and spontaneous mentalizing; explicit items scored from 0 to 2, and a single spontaneous question as a dichotomous yes/no variable. However, as the spontaneous question prompts participants to provide “the character’s thoughts, feelings and intentions when it applies to the question” (Dodell-Feder et al., 2013, p. 4) the implicit/explicit distinction is not clear cut. The coding scheme does not distinguish cognitive and affective, or first- and second-order attributions (indicated through low internal consistency; α = 0.54), signaling the need for a scoring system to support a more nuanced picture of mindreading (see Dodell-Feder et al., 2013, for some recommendations).

The original SST demonstrated sensitivity to variation among NT adults (scores ranged from 2 to 14 of 16 points), and concurrent validity with the Eyes Test and Interpersonal Reactivity Index fantasy subscale (IRI; Davis, 1983) supported it as a measure of the mindreading construct. Notably, recent evidence suggests that reading literary fiction can enhance performance on mindreading measures including the Eyes Test (Kidd and Castano, 2013), indicating that processes associated with fiction-engagement may prime the mindreading mechanism. In the original SST, participants completed all mindreading measures *after* reading, so future studies should vary task order to control for potential priming effects.

## Multidimensional Measures

### Narrative Fiction (Film)

Film stimuli enable researchers to present dynamic interactions (e.g., Golan et al., 2008; Barnes et al., 2009; Bazin et al., 2009), but can lack the range and complexity of ecological mindreading. Using actors to simulate social scenarios offers increased control over context and content variables. The Movie for the Assessment of Social Cognition (MASC; Dziobek et al., 2006), features four characters at a dinner party. A script development process (Field et al., 2001) generated realistic characters (displaying stable traits and transient states) and prominent themes are romance and friendship. Participants answer direct questions about the characters’ cognitive and affective mental states, requiring interpretation of vocal, physical and contextual information, alongside classic mindreading concepts such as false-beliefs, metaphor and *faux pas*.

In the validation study, the MASC converged with three extant mindreading measures: a basic emotion recognition task, the Eyes Test and Strange Stories Task (shortened). However, MASC scores predicted Strange Stories Task performance in participants with Asperger Syndrome, and emotion recognition in controls, which indicated that verbal strategies may have compensated for facial processing difficulties. The authors recommended future studies vary mental state complexity and part of the face focused on (eyes/mouth). Additionally, both groups performed at ceiling on the control questions, so future revisions should incorporate more challenging questions to account for other cognitive processes (Heavey et al., 2000; Dziobek et al., 2006). Notably, the MASC was more sensitive to group differences than the established measures, supported by a recent finding that participants with ASD showed impaired MASC, but not Eyes Test performance, when compared to participants with alexithymia (a condition characterized by impaired emotion recognition that often co-occurs with ASD; Oakley et al., 2016). This suggested that the emotion recognition deficit deemed characteristic of ASD may be due to alexithymia, highlighting the MASC’s sensitivity to selective deficits and diagnostic potential.

Versions of the MASC include the original German and dubbed English editions [dubbing did not interfere with participants’ task focus (Dziobek et al., 2006) and generally does not impact information processing (Koolstra et al., 2002)]. Generalizability and longevity may be limited, however, due to the contextually specific nature of mental state attributions (interactions may be better understood by similar age-groups to the characters, for example; Griffiths, 1997). This is, to some extent, also true for fictional prose. Moreover, in light of evidence that reading fiction can enhance mindreading, we have suggested that narrative-engagement may prime task performance. While task order was not reported for the MASC, similar effects have been found for television dramas (Black and Barnes, 2015), so future researchers should consider the potentially moderating effects of narrative-engagement processes when employing either fiction approach.

### Participative Interaction

Individuals not only observe—they interact with—the social world. Interactive approaches to measuring mindreading include a participative version of the *Empathic Accuracy Paradigm* (Ickes et al., 1990). Pairs of participants are covertly filmed waiting to participate in an experiment. After debriefing, they individually watch the footage back to identify their thoughts and feelings, and infer the mental states of their partner. Partner inferences are scored for accuracy. The procedure is socially valid, but contingent on individuals accurately articulating their own mental states (Cuff et al., 2014), and limited to the range of states naturally occurring in the context.

The advancement of virtual environment (VE) technology enables the construction of more complex interactive scenarios. The *Interactive Real World Task* (Spiers and Maguire, 2006) requires participants to retrospectively describe their thoughts during a driving simulation. The original study allowed researchers to observe patterns in *f*MRI data in relation to participants’ mentalizing. The task was designed to elicit spontaneous mindreading, however, direct questions and a coding system containing accuracy and complexity variables could facilitate a temporal view of explicit decoding and reasoning processes, whilst advancing knowledge of the neural network underlying mindreading.

Training is required prior to participation in VEs, however, this could prove a beneficial tradeoff for studying live, interactive mindreading whilst incorporating the range of variables available to fiction approaches. As with fiction tasks, processes associated with interpreting the narrative features of VEs may impact mindreading performance; research has shown that in-game storytelling enhances affective mindreading (e.g., Bormann and Greitemeyer, 2015). While this necessitates additional measures to control for individual differences in narrative-engagement, it also signifies the potential utility of VEs in interpersonal skills training. VE training offers greater ecological validity than previous tools (e.g., false-belief training; Parsons and Mitchell, 2002) and preliminary data from ASD groups indicates that it can improve both emotion recognition and mentalizing abilities (Kandalaft et al., 2013).

## Conclusion

Extant mindreading research has primarily focused on children, ASD, and clinical populations, and standard measures can suffer ceiling effects with NT adults. However, several classic tests have been adapted for research with adult cohorts. Tasks measuring specific mindreading dimensions such as emotion perception and cognitive mentalizing risk subtracting out key processes, particularly as social displays can be suppressed, and so interpreting mental states may require integration of verbal, physical and contextual information (McDonald et al., 2003). In contrast, complex, naturalistic approaches, including fiction-based and interactive tasks, reflect the multiplicity of ecological mindreading and speak to a multi-systems cognitive architecture (Christensen and Michael, 2016). We suggest, however, that researchers using fictional prose, film and VEs should consider the potentially moderating processes associated with narrative-engagement. As VEs have proven efficacious both in interpersonal skills development and studying the neural basis of mindreading, this may prove a worthwhile tradeoff. Multidimensional approaches are often resource-heavy and necessitate complex scoring systems to avoid compensatory strategies masking selective deficits. Therefore, we suggest the concomitant use of established multidimensional *and* simpler measures, to assess concurrent validity and probe mindreading variation both within and between participants. In this way, multidimensional stimuli need not problematize construct validity, but could prove fruitful to the development of multi-systems approaches, and studies of the neural architecture underlying mindreading in adulthood, which in turn may expand the wider social cognition literature.

## Author Contributions

FF: Concept, design, literature review (comments, suggestions), writing (comments, suggestions). RT: Writing, literature review.

## Conflict of Interest Statement

The authors declare that the research was conducted in the absence of any commercial or financial relationships that could be construed as a potential conflict of interest.

## References

[B1] Achim A. M. Guitton M. Jackson P. L. Boutin A. Monetta L. (2013). On what ground do we mentalize? Characteristics of current tasks and sources of information that contribute to mentalizing judgments. *Psychol. Assess.* 25 117–126. 10.1037/a0029137 22731676

[B2] Adolphs R. Baron-Cohen S. Tranel D. (2002). Impaired recognition of social emotions following amygdala damage. *J. Cogn. Neurosci.* 14 1264–1274. 10.1162/089892902760807258 12495531

[B3] Apperly I. A. Butterfill S. A. (2009). Do humans have two systems to track beliefs and belief-like states? *Psychol. Rev.* 116 953–970. 10.1037/a0016923 19839692

[B4] Apperly I. A. Samson D. Humphreys G. W. (2009). Studies of adults can inform accounts of theory of mind development. *Dev. Psychol.* 45 190–201. 10.1037/a0014098 19210001

[B5] Astington J. W. Dack L. A. (2008). “Theory of mind,” in *Encyclopedia of Infant and Early Childhood Development, 3* eds Haith M. M. Benson J. B. (San Diego, CA: Academic Press) 343–356.

[B6] Awasthi A. Mandal M. K. (2015). “Facial expressions of emotions: research perspectives,” in *Understanding Facial Expressions in Communication* eds Awasthi A. Mandal M. K. (Berlin: Springer) 1–18. 10.1007/978-81-322-1934-7

[B7] Back E. Apperly I. A. (2010). Two sources of evidence on the non-automaticity of true and false belief ascription. *Cognition* 115 54–70. 10.1016/j.cognition.2009.11.008 20060965

[B8] Barnes J. L. Lombardo M. V. Wheelwright S. Baron-Cohen S. (2009). Moral dilemmas film task: a study of spontaneous narratives by individuals with autism spectrum conditions. *Autism Res.* 2 148–156. 10.1002/aur.79 19575384

[B9] Baron-Cohen S. Jolliffe T. Mortimore C. Robertson M. (1997). Another advanced test of theory of mind: evidence from very high-functioning adults with autism or Asperger Syndrome. *J. Child Psychol. Psychiatry* 38 813–822. 10.1111/j.1469-7610.1997.tb01599.x 9363580

[B10] Baron-Cohen S. Leslie A. M. Frith U. (1985). Does the autistic child have a “theory of mind”? *Cognition* 21 37–46. 10.1016/0010-0277(85)90022-8 2934210

[B11] Baron-Cohen S. Wheelwright S. Hill J. Raste Y. Plumb I. (2001a). The “Reading the Mind in the Eyes” Test revised version: a study with normal adults, and adults with Asperger Syndrome or high-functioning autism. *J. Child Psychol. Psychiatry* 42 241–251. 10.1111/1469-7610.00715 11280420

[B12] Baron-Cohen S. Wheelwright S. Skinner R. Martin J. Clubley E. (2001b). The Autism-Spectrum Quotient (AQ): evidence from Asperger Syndrome/high-functioning autism, males and females, scientists and mathematicians. *J. Autism Dev. Disord.* 31 5–17. 10.1023/A:1005653411471 11439754

[B13] Bazin N. Brunet-Gouet E. Bourdet C. Kayser N. Falissard B. Hardy-Baylé M. (2009). Quantitative assessment of attribution of intentions to others in schizophrenia using an ecological video-based task: a comparison with manic and depressed patients. *Psychiatry Res.* 167 28–35. 10.1016/j.psychres.2007.12.010 19346006

[B14] Biele C. Grabowska A. (2006). Sex differences in perception of emotion intensity in dynamic and static facial expressions. *Exp. Brain Res.* 171 1–6. 10.1007/s00221-005-0254-0 16628369

[B15] Birch S. A. J. Bloom P. (2007). The curse of knowledge in reasoning about false beliefs. *Psychol. Sci.* 18 382–386. 10.1111/j.1467-9280.2007.01909.x 17576275

[B16] Black J. Barnes J. L. (2015). Fiction and social cognition: the effect of viewing award-winning television dramas on theory of mind. *Psychol. Aesthet. Creat. Arts* 9 355–494. 10.1037/aca0000031

[B17] Bloom P. German T. P. (2000). Two reasons to abandon the false belief task as a test of theory of mind. *Cognition* 77 25–31. 10.1016/S0010-0277(00)00096-2 10980256

[B18] Bormann D. Greitemeyer T. (2015). Immersed in virtual worlds and minds: effects of in-game storytelling on immersion, need satisfaction, and affective theory of mind. *Soc. Psychol. Personal. Sci.* 6 646–652. 10.1177/1948550615578177

[B19] Bull R. Phillips L. A. Conway C. A. (2008). The role of control functions in mentalizing: dual-task studies of theory of mind and executive function. *Cognition* 107 663–672. 10.1016/j.cognition.2007.07.015 17765214

[B20] Castano E. (2012). “Anti-social behavior in individuals and groups: an empathy-focused approach,” in *The Oxford Handbook of Personality and Social Psychology* eds Deux K. Snyder M. (New York, NY: Oxford University Press) 419–445.

[B21] Castelli F. Happé F. Frith U. Frith C. (2000). Movement and mind: a functional imaging study of perception and interpretation of complex intentional movement patterns. *NeuroImage* 12 314–325. 10.1006/nimg.2000.0612 10944414

[B22] Christensen W. Michael J. (2016). From two systems to a multi-systems architecture for mindreading. *New Ideas Psychol.* 40 48–64. 10.1016/j.hewideapsych.2015.01.003

[B23] Cuff B. M. P. Brown S. J. Taylor L. Howat D. J. (2014). Empathy: a review of the concept. *Emot. Rev.* 0 1–10. 10.1016/j.newideapsych.2015.01.003

[B24] Davis M. H. (1983). Measuring individual differences in empathy: evidence for a multidimensional approach. *J. Pers. Soc. Psychol.* 44 113–126. 10.1037/0022-3514.44.1.113

[B25] Dennett D. (1978). Beliefs about beliefs. *Behav. Brain Sci.* 1 568–570. 10.1017/S0140525X00076664

[B26] Dodell-Feder D. Koster-Hale J. Bedny M. Saxe R. (2011). fMRI item analysis in a theory of mind task. *Neuroimage* 55 705–712. 10.1016/j.neuroimage.2010.12.040 21182967

[B27] Dodell-Feder D. Lincoln S. H. Coulson J. P. Hooker C. I. (2013). Using fiction to assess mental state understanding: a new task for assessing theory of mind in adults. *PLoS ONE* 8:e81279 10.1371/journal.pone.0081279 PMC3820595 24244736

[B28] Duval C. Piolino P. Bejanin A. Eustache F. Desgranges B. (2010). Age effects on different components of theory of mind. *Consci. Cogn.* 20 627–642. 10.1016/j.concog.2010.10.025 21111637

[B29] Dziobek I. Fleck S. Kalbe E. Rogers K. Hassenstab J. Brand M. (2006). Introducing MASC: a movie for the assessment of social cognition. *J. Autism Dev. Dis.* 36 623–636. 10.1007/s10803-006-0107-0 16755332

[B30] Ekman P. Friesen W. V. (1971). Constants across culture in the face and emotion. *J. Pers. Soc. Psychol.* 17 124–129. 10.1037/h0030377 5542557

[B31] Ekman P. Friesen W. V. (1974). “Nonverbal behavior and psychopathology,” in *The Psychology of Depression* eds Friedman R. J. Katz M. M. (Washington, DC: Winston & Sons) 203–224.

[B32] Ekman P. Friesen W. V. (1976). *Pictures of Facial Affect.* Palo Alto, CA: Consulting Psychologists Press.

[B33] Epley N. Morowedge C. K. Keysar B. (2004). Perspective taking in children and adults: equivalent egocentricism but differential correction. *J. Exp. Soc. Psychol.* 40 760–768. 10.1016/j.jesp.2004.02.002

[B34] Fett A. J. Viechtbauer W. Dominguez M. Penn D. L. van Os J. Krabbendam L. (2011). The relationship between neurocognition and social cognition with functional outcomes in schizophrenia: a meta-analysis. *Neurosci. Biobehav. Rev.* 35 573–588. 10.1016/j.neubiorev.2010.07.001 20620163

[B35] Field S. Meyer A. Witte G. (2001). *Drehbuchschreiben Fur Fersehen Und Film.* Munchen: Ullstein Verlag.

[B36] Frank M. G. Svetieva E. (2015). “Microexpressions and deception,” in *Understanding Facial Expressions in Communication* eds Awasthi A. Mandal M. K. (Berlin: Springer) 227–242. 10.1007/978-81-322-1934-7

[B37] Frith C. D. Corcoran R. (1996). Exploring ‘theory of mind’ in people with schizophrenia. *Psychol. Med.* 26 521–553. 10.1017/S0033291700035601 8733211

[B38] Golan O. Baron-Cohen S. Golan Y. (2008). The “Reading the Mind in Films” task [Child Version]: complex emotion and mental state recognition in children with and without autism spectrum conditions. *J. Autism Dev. Dis.* 38 1534–1541. 10.1007/s10803-007-0533-7 18311514

[B39] Gray K. Jenkins A. C. Heberlein A. S. Wegner D. M. (2010). Distortions of mind perception in psychopathology. *Proc. Natl. Acad. Sci. U.S.A.* 108 477–479. 10.1073/pnas.1015493108 21187372 PMC3021012

[B40] Gregory C. Lough S. Stone V. Erzinclioglu S. Martin L. Baron-Cohen S. (2002). Theory of mind in patients with frontal variant frontotemporal dementia and Alzheimer’s disease: theoretical and practical implications. *Brain* 125 752–764. 10.1093/brain/awf079 11912109

[B41] Griffiths P. (1997). *What Emotions Really are: The Problem of Psychological Categories.* Chicago: University of Chicago Press.

[B42] Guastella A. J. Hermens D. F. Van Zweiten A. Naismith S. L. Lee R. S. C. Cacciotti-Saija C. (2013). Social cognitive performance as a marker of positive psychotic symptoms in young people seeking help for mental health problems. *Schizophr. Res.* 149 77–82. 10.1016/j.schres.2013.06.006 23845388

[B43] Halberstadt A. G. Dennis P. A. Hess U. (2011). The influence of family expressiveness, individuals’ own emotionality, and self-expressiveness on perceptions of others’ facial expressions. *J. Nonverbal Behav.* 35 35–50. 10.1007/s10919-010-0099-5

[B44] Happé F. Brownell H. Winner E. (1999). Acquired ’theory of mind’ impairments following stroke. *Cognition* 70 211–240. 10.1016/S0010-0277(99)00005-0 10384736

[B45] Happé F. G. E. (1994). An advanced test of theory of mind: understanding of story characters’ thoughts and feelings by able autistic, mentally handicapped, and normal children and adults. *J. Autism. Dev. Disord.* 24 129–154. 10.1007/BF02172093 8040158

[B46] Happé F. G. E. (1995). The role of age and verbal ability in the theory of mind task performance of subjects with autism. *Child Dev.* 66 843–855. 10.1111/j.1467-8624.1995.tb00909.x 7789204

[B47] Happé F. G. E. Winner E. Brownell H. (1998). The getting of wisdom: theory of mind and old age. *Dev. Psychol.* 34 358–362. 10.1037/0012-1649.34.2.358 9541787

[B48] Heavey L. Phillips W. Baron-Cohen S. Rutter M. (2000). The awkward moments test: a naturalistic measure of social understanding in autism. *J. Autism Dev. Dis.* 30 225–236. 10.1023/A:1005544518785 11055458

[B49] Hemingway E. (2003). *The End of Something. In Our Time.* New York, NY: Scribner.

[B50] Heyes C. M. Frith C. D. (2014). The cultural evolution of mindreading. *Science* 344 1357–1363. 10.1126/science.1243091 24948740

[B51] Ickes W. Stinson L. Bissonnette V. Garcia S. (1990). Naturalistic social cognition: empathic accuracy in mixed-sex dyads. *J. Pers. Soc. Psychol.* 59 730–732. 10.1037/0022-3514.59.4.730

[B52] Johnson D. R. (2012). Transportation into a story increases empathy, prosocial behavior, and perceptual bias toward fearful expressions. *Pers. Individ. Differ.* 52 150–155. 10.1016/j.paid.2011.10.005

[B53] Kalbe E. Schlegel M. Sack A. T. Nowak D. A. Dafotakis M. Bangard C. (2010). Dissociating cognitive from affective theory of mind: a TMS study. *Cortex* 46 769–780. 10.1016/j.cortex.2009.07.010 19709653

[B54] Kandalaft M. R. Didehbani N. Krawczyk D. C. Allen T. T. Chapman S. B. (2013). Virtual reality social cognition training for young adults with high-functioning autism. *J. Autism. Dev. Disord.* 43 33–44. 10.1007/s10803-012-1544-6 PMC3536992 22570145

[B55] Kanske P. Böckler A. Trautwein F. Singer T. (2015). Dissecting the social brain: introducing the EmpaToM to reveal distinct neural networks and brain-behaviour relations for empathy and Theory of Mind. *NeuroImage* 122 6–19. 10.1016/j.neuroimage.2015.07.082 26254589

[B56] Kidd D. C. Castano E. (2013). Reading literary fiction improves theory of mind. *Science* 342 377–380. 10.1126/science.1239918 24091705

[B57] Koolstra C. Peeters A. Spinhof H. (2002). The pros and cons of dubbing and subtitling. *Eur. J. Commun.* 17 325–354. 10.1177/0267323102017003694

[B58] Le Bouc R. Lenfant P. Delbeuck X. Ravasi L. Lebert F. Semah F. (2012). My belief or yours? Differential theory of mind deficits in frontotemporal dementia and Alzheimer’s disease. *Brain* 135 3026–3038. 10.1093/brain/aws237 23065791

[B59] Lind S. E. Bowler D. M. (2009). Language and theory of mind in autism spectrum disorder: the relationship between complement syntax and false belief task performance. *J. Autism. Dev. Disord.* 39 929–937. 10.1007/s10803-009-0702-y 19205856

[B60] Matsumoto D. LeRoux J. Wilson-Cohn C. Raroque J. Kooken K. Ekman P. (2000). A New test to measure emotion recognition ability: matsumoto and Ekman’s Japanese and Caucasian Brief Affect Recognition Test (JACBART). *J. Nonverbal Behav.* 24 179–209. 10.1023/A:1006668120583

[B61] Maylor E. A. Moulson J. M. Muncer A. M. Taylor L. A. (2002). Does performance on theory of mind tasks decline in old age? *Br. J. Psychol.* 93 465–485. 10.1348/000712602761381358 12519529

[B62] McDonald S. Flanagan S. Rollins J. Kinch J. (2003). TASIT: a new clinical tool for assessing social perception after traumatic brain injury. *J. Head Trauma Rehabil.* 18 219–238. 10.1097/00001199-200305000-00001 12802165

[B63] Mitchell P. Robinson E. J. Isaaca J. E. Nye R. M. (1996). Contamination in reasoning about false belief: an instance of realist bias in adults but not children. *Cognition* 59 1–21. 10.1016/0010-0277(95)00683-4 8857469

[B64] Mitchell R. L. C. Phillips L. H. (2015). The overlapping relationship between emotion perception and theory of mind. *Neuropsychologia* 70 1–10. 10.1016/j.neuropsychologia.2015.02.018 25687032

[B65] Nichols S. Stich S. P. (2003). *Mindreading: An Integrated Account of Pretence, Self-Awareness, and Understanding Other Minds.* Oxford: Clarendon Press.

[B66] Oakley B. F. M. Brewer R. Bird G. Catmur C. (2016). ‘Theory of mind’ is not theory of emotion: a cautionary note on the Reading the Mind in the Eyes Test. *J. Abnorm. Psychol.* 125 818–823. 10.1037/abn0000182 27505409 PMC4976760

[B67] Onishi K. H. Baillargeon R. (2005). Do 15-month-old infants understand false beliefs? *Science* 308 255–258. 10.1126/science.1107621 15821091 PMC3357322

[B68] Paal T. Bereczkei T. (2007). Adult theory of mind, cooperation, machiavellianism: the effect of mindreading on social relations. *Pers. Individ. Differ.* 43 541–551. 10.1016/j.paid.2006.12.021

[B69] Parsons S. Mitchell P. (2002). The potential of virtual reality in social skills training for people with autistic spectrum disorders. *J. Intellect. Disabil. Res.* 46 430–443. 10.1046/j.1365-2788.2002.00425.x 12031025

[B70] Perner J. Ruffman T. (2005). Infants’ insight into the mind: how deep? *Science* 308 214–216. 10.1126/science.1111656 15821079

[B71] Perner J. Wimmer H. (1985). “John thinks that Mary thinks that”: attribution of second-order beliefs by 5- to 10-year old children. *J. Exp. Child Psychol.* 39 437–471. 10.1016/0022-0965(85)90051-7

[B72] Pessoa L. (2008). On the relationship between emotion and cognition. *Nat. Rev. Neurosci.* 9 148–158. 10.1038/nrn2317 18209732

[B73] Poletti M. Enrici I. Adenzatio M. (2012). Cognitive and affective theory of mind in neurodegenerative diseases: neuropsychological, neuroanatomical and neurochemical levels. *Neurosci. Biobehav. Rev.* 36 2147–2164. 10.1016/j.neubiorev.2012.07.004 22819986

[B74] Premack D. Woodruff G. (1978). Does the chimpanzee have a theory of mind? *Behav. Brain Sci.* 1 515–526. 10.1017/S0140525X00076512

[B75] Rutherford M. D. Baron-Cohen S. Wheelwright S. (2002). Reading the mind in the voice: a study with normal adults and adults with Asperger syndrome and high functioning autism. *J. Autism. Dev. Disord.* 32 189–194. 10.1023/A:1015497629971 12108620

[B76] Saxe R. Kanwisher N. (2003). People thinking about thinking people: the role of the temporo-parietal junction in “theory of mind”. *Neuroimage* 19 1835–1842. 10.1016/S1053-8119(03)00230-1 12948738

[B77] Saxe R. Wexler A. (2005). Making sense of another mind: the role of the right temporo-parietal junction. *Neuropsychologia* 43 1391–1399. 10.1016/j.neuropsychologia.2005.02.013 15936784

[B78] Schaafsma S. M. Pfaff D. W. Spunt R. P. Adolphs R. (2015). Deconstructing and reconstructing theory of mind. *Trends Cogn. Sci.* 19 65–72. 10.1016/j.tics.2014.11.007 25496670 PMC4314437

[B79] Senju A. Southgate V. White S. Frith U. (2009). Mindblind eyes: an absence of spontaneous theory of mind in Asperger syndrome. *Science* 325 883–885. 10.1126/science.1176170 19608858

[B80] Shamay-Tsoory S. G. Aharon-Peretz J. (2007). Dissociable prefrontal networks for cognitive and affective theory of mind: a lesion study. *Neuropsychologia* 45 3054–3067. 10.1016/j.neuropsychologia.2007.05.021 17640690

[B81] Siegal M. Beattie K. (1991). Where to look first for children’s knowledge of false beliefs. *Cognition* 38 1–12. 10.1016/0010-0277(91)90020-5 2015753

[B82] Spiers H. J. Maguire E. A. (2006). Spontaneous mentalizing during an interactive real world task: an fMRI study. *Neuropsychologia* 44 1674–1682. 10.1016/j.neuropsychologia.2006.03.028 16687157

[B83] Spunt R. P. Adolphs R. (2014). Validating the why/how contrast for functional mri studies of theory of mind. *Neuroimage* 99 301–311. 10.1016/j.neuroimage.2014.05.023 24844746 PMC4111963

[B84] Sullivan K. Zaitchik D. Tager-Flusberg H. (1994). Preschoolers can attribute second-order beliefs. *Dev. Psychol.* 30 395–402. 10.1037/0012-1649.30.3.395

[B85] Sze J. A. Goodkind M. S. Gyurak A. Levenson R. W. (2012). Aging and emotion recognition: not just a losing matter. *Psychol. Ageing* 27 940–950. 10.1037/a0029367 PMC3746016 22823183

[B86] Wang J. J. Miletich D. D. Ramsey R. Samson D. (2014). Adults see vision to be more informative than it is. *Q. J. Exp. Psychol.* 67 2279–2292. 10.1080/17470218.2014.915331 PMC4226390 24853581

[B87] Wellman H. M. Cross D. Watson J. (2001). Meta-analysis of theory-of-mind development: the truth about false belief. *Child Dev.* 72 655–684. 10.1111/1467-8624.00304 11405571

[B88] Wimmer H. Perner J. (1983). Beliefs about beliefs: representation and constraining function of wrong beliefs in young children’s understanding of deception. *Cognition* 13 103–128. 10.1016/0010-0277(83)90004-5 6681741

[B89] Winner E. Brownell H. Happé F. Blum A. Pincus D. (1998). Distinguishing lies from jokes: theory of mind deficits and discourse interpretation in right hemisphere brain-damaged patients. *Brain Lang.* 62 89–106. 10.1006/brln.1997.1889 9570881

